# Success in Parenthood: The In Vitro Maturation Breakthrough in Infertility

**DOI:** 10.7759/cureus.57486

**Published:** 2024-04-02

**Authors:** Namrata Choudhary, Jarul Shrivastava, Akash More, Ashish Anjankar, Deepti Shrivastava

**Affiliations:** 1 Clinical Embryology, Datta Meghe Institute of Higher Education and Research, Wardha, IND; 2 Biochemistry, Datta Meghe Institute of Higher Education and Research, Wardha, IND; 3 Obstetrics and Gynecology, Datta Meghe Institute of Higher Education and Research, Wardha, IND

**Keywords:** introcytoplasmic sperm injection (icsi), asthenozoospermia, polycystic ovary syndrome (pcos), infertility and art, in vitro maturation

## Abstract

This case report revolves around a 37-year-old woman and her 39-year-old husband, who have been married for seven years and were seeking treatment for infertility. The husband has been diagnosed with asthenozoospermia for the past six years and has been on continued medication, and the woman has been diagnosed with polycystic ovarian syndrome (PCOS). To improve fertility outcomes, this case report enlightens the treatment and medical strategy for people with PCOS. Treatment included low-dose ovarian stimulation for the removal of immature eggs, and then in vitro maturation (IVM) of those oocytes was done. Later, intracytoplasmic sperm injection (ICSI) was performed to form the blast. The formed blasts were later cryopreserved till embryo transfer. This case report highlights the importance of preventing the risk of ovarian hyperstimulation syndrome (OHSS) in patients with PCOS.

## Introduction

Polycystic ovary syndrome (PCOS) is a hormonal disorder of women of reproductive age group and presents as irregular menstrual cycles, excess androgens, or polycystic ovaries [1]. Excess androgens or hyperandrogenism can lead to symptoms like acne, hirsutism, or baldness in females [2]. PCOS is a very common cause of female infertility due to the failure of the ovaries to release eggs. In vitro fertilization (IVF) can be the savior for such patients [3]. The ovarian stimulation involved in IVF can pose serious problems in PCOS patients, like ovarian hyperstimulation syndrome (OHSS) [4]. To combat that, in vitro maturation (IVM) of oocytes can be done while using stimulation for a very short period [5].

For women with polycystic ovaries (PCO), in vitro maturation is a suitable stimulation protocol [6]. These individuals face a higher risk of experiencing ovarian hyperstimulation syndrome (OHSS), which is a serious condition [4]. IVM can also be beneficial for women recovering from cancer [7]. IVM can be added to in vitro fertilization (IVF) protocols for various reasons. Since very little gonadotropin supplementation is required, IVM is a good alternative in IVF attempts to reduce the risk of OHSS [8]. IVM has also gained popularity in fertility preservation. Moreover, in infertility with ovarian resistance to follicle-stimulating hormone (FSH), IVM can be a significant approach for treating it [9].

## Case presentation

Patient information

This case report focuses on an Indian couple who came to our center to fulfill their dream of becoming parents despite having infertility. PCOS has been diagnosed in 37-year-old women for the last five years. Both were working in a private company. Both individuals were counseled regarding all procedures, benefits, and risks. The center obtained informed consent from them.

Medical history

The couple had undergone multiple cycles of intrauterine insemination (IUI) in the past but were not able to achieve pregnancy outcomes. A complete history was taken, and routine investigations were advised before a treatment plan was formulated for them. At first glance, the case appeared straightforward, but during further investigations, it was observed that the woman was suffering from a slower follicular growth with many smaller follicles, so an in vitro Maturation (IVM) was suggested for them. On semen analysis, the sperm count was 35 million partners with 15% motility, which falls under the abnormal range according to WHO guidelines. The patient visited our center in 2021 for further testing and treatment.

Physical examination and hormonal profile

The patient was physically healthy and in no acute distress. Heart rate, blood pressure, and respiration rate all appear to be normal. Table 1 and Table 2 show both partners hormonal profiles.

**Table 1:** Hormonal profile of the female partner

Parameters	Lab value	Reference value
Testosterone ng/mL	2.6	0.15-0.7 ng/mL
Luteinizing hormone mIU/mL	17.3	5-15 mIU/mL
Estradiol pg/mL	33.77	25-450 pg/mL
Progesterone ng/mL	0.31	1-8 ng/mL
Follicule-stimulating hormone mIU/mL	2.87	3.5-12.5 mIU/mL
Anti-Mullerian hormone ng/mL	7.76	1.0-4.0 ng/mL
Prolactin ng/mL	14.0	2-29 ng/mL

**Table 2:** Hormonal profile of the male partner

Parameters	Lab values	Reference value
Testosterone (ng/dL)	295	300-1000 ng/dL
Follicle-stimulating hormone (mIU/mL)	1.48	1.5-12.4 mIU/mL
Luteinizing Hormone (mIU/mL)	3.04	1.7-8.6 mIU/mL
Prolactin (ng/mL)	1.83	2-18 ng/mL
Thyroid-stimulating hormone (mIU/mL)	2.4	0.4-4.0 mIU/L

Ultrasound findings

The uterus appears normal with a normal texture. When the patient was examined with 2D ultrasonography, images of the patient's left and right ovaries showed no big follicles. The right ovary had 14 small follicles, and the left ovary had 12 small follicles.

Treatment

**Figure 1:**
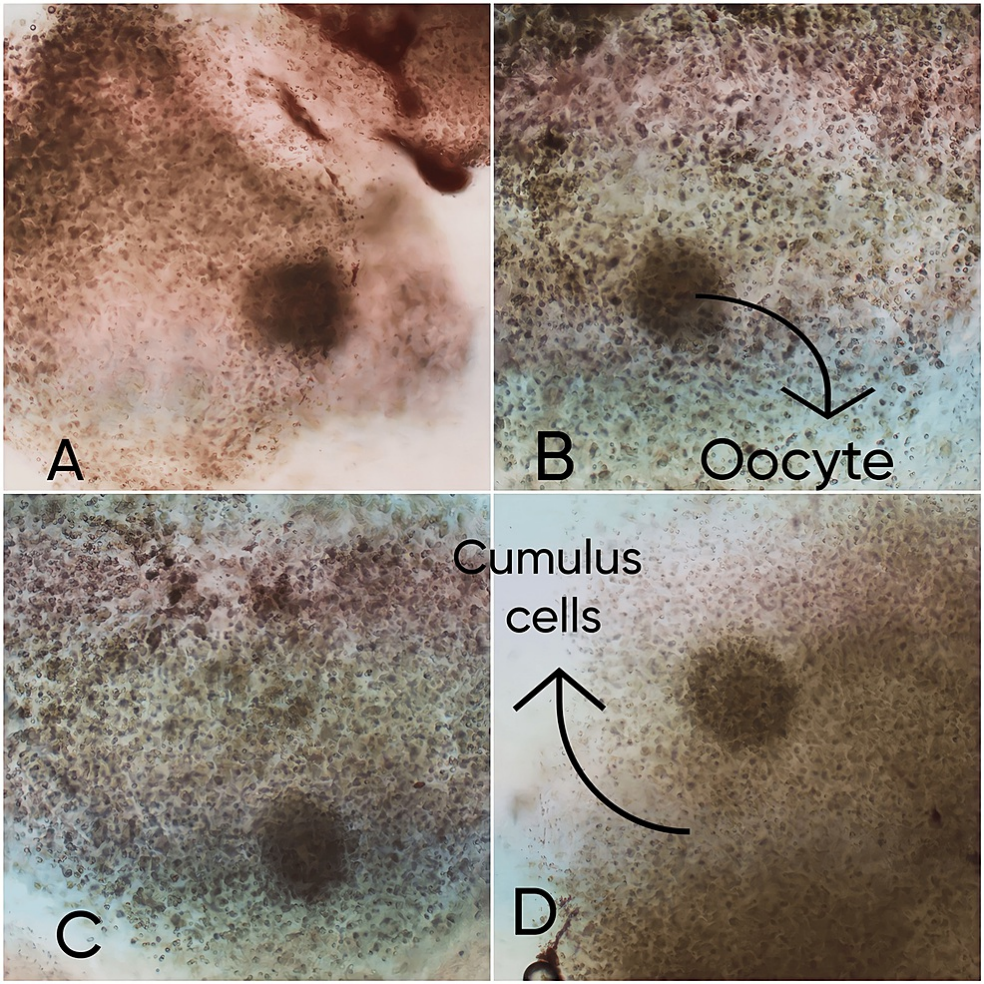
Enclosed oocytes retrieved from small antral follicles

To Initiate the IVF cycle, the woman was given 100 IU/day of FSH for only three days then, followed by the trigger of hCG 10000 IU, which was administered 36 hours before the ovum pick-up. Oocyte growth and maturity were observed every other day of stimulation. On the day of oocyte pick-up (OPU), a total of 10 immature oocytes were recovered. Figure 1 shows oocytes with cumulus cells.

**Figure 2:**
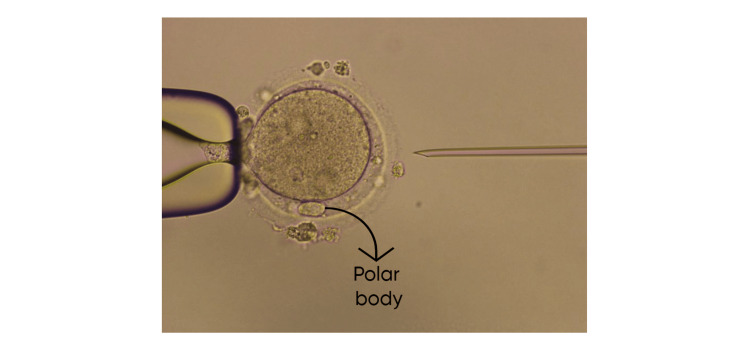
Metaphase II (MII) oocyte after in vitro maturation; polar body can be seen at 6 o'clock

Among retrieved oocytes, three transform into metaphase II (MII) oocytes, as shown in Figure 2. 

**Figure 3:**
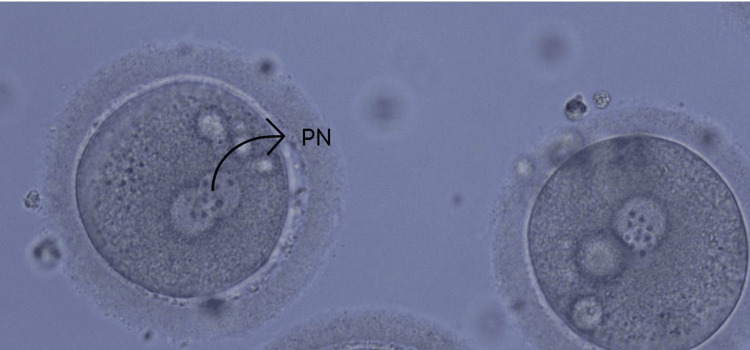
Pronuclei (PN) formed after intracytoplasmic sperm injection of metaphase II oocytes

These oocytes were fertilized using intracytoplasmic sperm injection (ICSI), as shown in Figure 3.

**Figure 4:**
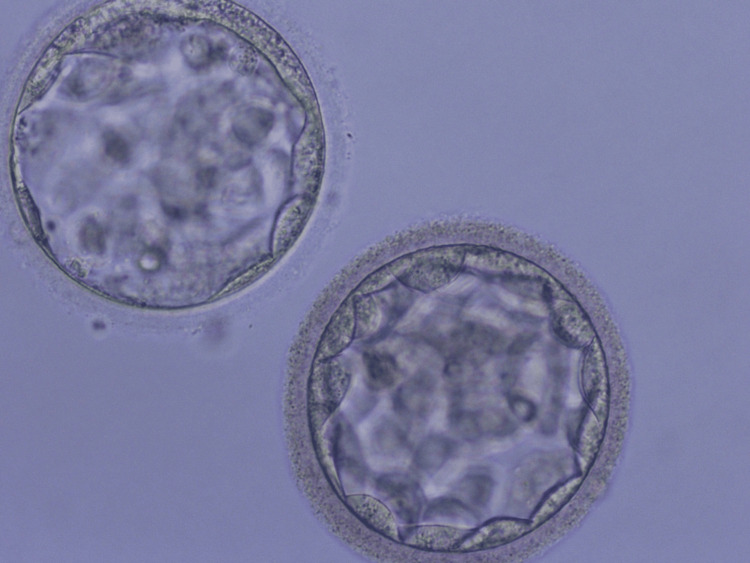
Blastocyst formed from in vitro maturation (IVM) oocytes

Fertilized oocytes transformed into two blastocysts of grade 4BB and 4BC quality as shown in Figure 4 and vitrified on single cryoprotective device. The couple was informed about the number and quality of the embryos.

Endometrial preparation protocol

The endometrial preparation protocol included low-dose exogenous estradiol 2 mg daily, metformin 250 mg, and cabergoline 5 mg twice a day. L-arginine sachets were also recommended to thicken the endometrial lining starting after oocyte retrieval. Progesterone was initiated six days before the scheduled embryo transfer, with an endometrial thickness measuring 7.8 mm on the transfer day. Followed by 2D5, the frozen embryo was thawed and embryo transferred. By using a commercially available thawing kit, the embryos were thawed.

Follow-up

After the embryo transfer, the patient was suggested to take adequate rest and avoid any type of stressful activity, which can cause stress to her, followed by calcium supplement for calcium intake, 5 mg of prednisolone, multivitamins, iron supplements, and injectable progesterone. After 14 days, the patient was asked for a urine pregnancy test (UPT). The UPT showed a positive result with a value of β-hCG-262 mlU/ml, a few weeks later, during a follow-up USG, the fetus was seen with normal parameters. The physician recommended that the patient maintain their medication.

## Discussion

PCOS is the cause of infertility in around 70% to 80% of women, as is evident from various studies [10]. Moreover, recurrent miscarriages are a common occurrence in women with PCOS [11]. Opting for IVF for such patients is always a good choice, but it can lead to complications like OHSS in PCOS patients. In vitro maturation is a safer alternative, preventing OHSS by maturing eggs outside the body [5]. A retrospective study by Shalom et al. compared treatment outcomes of IVM with IVF in PCOS patients. They concluded that in PCOS patients, IVM can replace conventional IVF [12]. Similar results were concluded by Shavit et al. [13].

In IVM, there is less use of hormones, which reduces the risk of OHSS, and it is also cost-effective. However, it's technically challenging rates [14]. Although the success rates of IVM are not higher than those of conventional methods, it can still be opted out because of its safer approach [15]. Child et al. [8] compared unstimulated IVM of oocytes with that of stimulated in vitro fertilization (IVF) in women with polycystic ovaries. IVF resulted in more mature oocytes and embryos compared to IVM, with higher implantation rates but also a higher risk of ovarian hyperstimulation syndrome (OHSS). However, IVM showed comparable pregnancy and live birth rates to IVF, suggesting it is a promising alternative for patients with polycystic ovaries opting for assisted conception treatment. In a study by Vuong et al., they used C-type natriuretic peptide-supplemented medium-assisted pre-fertilization in vitro maturation (CAPA-IVM), a biphasic culture system. Results showed more oocytes reaching maturity, higher embryo quality, and significantly better clinical pregnancy rates. Live births were reported for the first time with CAPA-IVM, indicating its promise as a safer and more effective fertility treatment for PCOM patients [16]. According to Chian et al., FSH or luteinizing hormone (LH) priming has shown benefits in oocyte maturation before retrieval of immature oocytes [17]. Likewise, Junk et al. presented an improved method for IVM of oocytes in women with PCOS by following protocols that included FSH priming, blastocyst culture, IVM, and hormone replacement therapy. It shows similar clinical pregnancy rates to in vitro fertilization [18].

## Conclusions

The case presented shows that the transfer of cryopreserved and thawed embryos from the IVM oocytes in PCOS patients can be possible and may result in pregnancy and live birth. Since endometrial receptivity may be poor in IVM cycles, using IVM oocytes and transferring thawed embryos in artificial cycles may offer an option to improve implantation rates. This approach could be a useful way to assess the development of IVM oocytes well and the results of frozen and thawed embryos from these oocytes. It may be important for physicians who use a method called IVM to help people preserve their fertility.
